# Vaspin promotes chondrogenic differentiation of BMSCs via Akt activation in osteoarthritis

**DOI:** 10.1186/s12891-022-05295-9

**Published:** 2022-04-11

**Authors:** Junfeng Wang, Keshi Zhang, Shaolong Zhang, Zhenpeng Guan

**Affiliations:** 1 Department of Orthopedics, Peking University International Hospital, Beijing, 102206 China; 2 Department of Orthopedics, Peking University Shougang Hospital, Beijing, 100144 China

**Keywords:** Vaspin, Bone mesenchymal stem cells, Chondrocyte, Akt

## Abstract

**Background:**

The aim of this study was to investigate the role of Vaspin on the chondrogenic differentiation of bone mesenchymal stem cells (BMSCs), and its effect on chondrocyte survival and ECM secretion. We also assessed whether the Akt activation participates in these processes.

**Methods:**

In vivo, ﻿immunohistochemistry was used to examine the positive rate of the protein expressions of Akt in Wistar rat articular cartilage and subchondral bone after Vaspin intraperitoneal injection for 14 days. In vitro, we isolated and expanded BMSCs from Wistar rats, and further cultured BMSCs as pellets in a chondrogenic-differentiation medium supplemented with different concentrations of Vaspin. After 21 days, the pellets were processed for cell counting kit assay. The mRNA level of Akt, SOX9 and COL2A1 in the pellets were investigated using quantitative Real-Time polymerase chain reaction, and the protein level of COMP was detected using western blot.

**Results:**

During the chondrogenic differentiation of BMSCs, Vaspin promoted the chondrogenic differentiation of BMSCs and chondrocyte survival by activating the Akt pathway. These effects were significantly reduced by treatment with an Akt inhibitor. Moreover, Vaspin promoted chondrogenic differentiation of BMSCs by increasing the expression of markers in cartilage formation and extracellular matrix secretion. Furthermore, our study also found that Vaspin could increase Akt expression in cartilage cavities and subchondral bone in vivo.

**Conclusion:**

These findings demonstrate that Vaspin can promote the chondrogenic differentiation of BMSCs and chondrocyte survival via Akt activation. Our study provides new insights into the potential ability of Vaspin to ameliorate the chondrogenic differentiation of BMSCs and chondrocyte survival in OA.

**Supplementary Information:**

The online version contains supplementary material available at 10.1186/s12891-022-05295-9.

## Background

Osteoarthritis (OA) is the most common degenerative joint disease characterized by cartilage degeneration, subchondral bone sclerosis, osteophyte and synovitis formation [1]. The complex pathogenesis of OA involves mechanical, inflammatory and metabolic factors, which ultimately lead to joint degeneration and structural destruction in articular cartilage and subchondral bone [2].

During the formation of OA, cartilage composition gradually changes and loses its integrity, in which the most critical changes are chondrocyte metabolic alterations and apoptosis increasing abnormally, which experience a pathological shift of metabolic homeostasis and cartilage remodeling [3–5]. Mesenchymal stem cells (MSCs) originating from bone marrow are multilineage cells with the ability to self-renew, described as a population of progenitor cells capable of osteogenic, chondrogenic and adipogenic differentiation, which play key roles in tissue healing and regenerative medicine [6, 7]. Therefore, the change and its molecular mechanism of bone marrow mesenchymal stem cells (BMSCs) differentiation into chondrocytes, and chondrocyte metabolic alterations are important research targets for OA.

Recent studies have shown that adipokines are involved in the initiation and progression of OA, which could affect the metabolism of osteoblasts, osteoclasts and chondrocytes through a variety of signaling pathways, and participate in the internal balance of bone and cartilage [8]. Vaspin is a beneficial adipokine, which could counteract insulin-resistance and reduces inflammatory processes in obesity and other diseases [9, 10]. Our previous study has found that Vaspin is abnormally expressed in the OA bone marrow microenvironment, and can affect the proliferation and survival of BMSCs by regulating the PI3K/Akt signaling pathway [11]. However, whether Vaspin can contribute to attenuate chondrocyte dysfunction and extracellular matrix (ECM) degradation, thereby participate in the metabolic imbalance of cartilage in OA, there is no relevant report.

﻿In the current study, we aimed to investigate the role of Vaspin on the chondrogenic differentiation of BMSCs and chondrocyte survival, and its effect on ECM secretion. We also assessed whether the Akt activation participates in these processes.

## Materials and methods

### Animals

Eight-week-old healthy male Wistar rats weighing 180–220 g were purchased from Beijing HFK Bioscience Co. Ltd. (Beijing, China). The rats were placed in a room with controlled temperature conditions (21–22 °C) and lighting (12 h light/dark cycle), with access to sterile food and water. After 1 week of acclimatization, the rats were divided randomly into two groups: no Vaspin injection group (*n* = 6, intraperitoneal injection with the same amount of normal saline) and Vaspin injection group (*n* = 6, Vaspin intraperitoneal injection at a dose of 1 μg/kg/day for 14 days). Rat recombinant Vaspin protein was purchased from Abbexa (Cambridge, UK). After 14 days, articular cartilage with subchondral bone in distal femur was isolated for the subsequent histological experiments. Ethical approval was received from the ethics committee of Peking University International Hospital (2018–068(BMR)). We have complied with the Guide for the Care and Use of Laboratory Animals, published by the United States National Institutes of Health (2011). Animal experiments were conducted in accordance with the internationally accepted ethical guidelines.

### Rat BMSCs culture and chondrogenic differentiation

According to the manufacturer’s instructions, Wistar rat BMSCs at passage 2 (RAWMX-01001, Cyagen Biosciences Inc., China) were cultured in OriCell MSC growth medium supplemented with 10% fetal bovine serum, 1% glutamine, and 1% penicillin-streptomycin (RAWMX-90011, all reagents from Cyagen Biosciences Inc.). The medium was changed every other day. The cells were passaged after reaching 80–90% confluency. 5 × 10^6^ cell at passage 3–5 were harvested and centrifuged at 500 g for 3 min in a 15 ml centrifuge tube to generate a cell pellet. Cell pellet was incubated for 24 h with the maintenance medium. The medium was changed to chondrogenic differentiation medium (RAWMX-9004, Cyagen Biosciences Inc.) the next day, which was supplemented with 100 μM L-ascorbic acid, 100 μM sodium pyruvate, 100 μM L-proline, 100 nM dexamethasone, 1% ITS and 10 ng/mL TGF-β3 (all provided by Cyagen Biosciences Inc.). According to different groups, cell pellets were fed every 2 days with freshly made chondrogenic differentiation medium with different concentrations of Vaspin (0 ng/ml, 150 ng/ml and 300 ng/ml) for a total of 21 days. After chondrogenic differentiation and incubation with Vaspin, the cell pellets were used for further molecular experiments.

### Cell counting kit (CCK-8) assay

Chondrocyte proliferation was measured using CCK-8 assay (Dojindo, Japan). Briefly, after chondrogenic differentiation for 21 days (no Vaspin treatment group), the cell pellets were digested in 0.2% collagenase II for 1 h at 37 °C to harvest chondrocytes. We seeded chondrocytes in a 96-well plate at a density of 5000 cells/well for 24 h to achieve cell synchronization. Then, the cells were treated with 0 ng/ml Vaspin, 150 ng/ml Vaspin, and 150 ng/ml Vaspin in combination with 8 μmol/L Akt specific inhibitors API-2 for 24 h, respectively. After Vaspin treatment, 10 μl CCK-8 solution was added to each well, and the plates were incubated for 3 h at 37 °C. The optical density of each well was measured using a microplate reader (Thermo Fisher Scientific, USA) at a wavelength of 450 nm.

### RNA extraction and quantitative real-time polymerase chain reaction (qRT-PCR)

Total RNA was extracted from cell pellets at the end of week 3 using Trizol reagent (Thermo Fisher Scientific, USA), and reverse-transcribed to cDNA with RT reagent kit (Promega, USA), according to the manufacturer’s instructions. The qRT-PCR was performed with SYBR Green Realtime PCR Master Mix (Toyobo, Japan) according to the manufacturer’s protocol. The Real-time fluorescence quantitative PCR instrument was Mx3000P QPCR Systems (Agilent Technology Inc., USA) in this study. The specific qRT-PCR reaction system used in this study were as follow: pre-denaturation at 95 °C for 2 min, followed by 40 cycles of denaturation at 95 °C for 10s and annealing at 60 °C for 30 s and extension at 72 °C for 30 s. Primers sequences for Akt, SOX9, COL2A1 and GAPDH were listed in Table 1. The gene expression was analyzed by 2^−ΔΔCt^ method using GAPDH as the internal control. All the experiments were repeated independently three times.

**Table 1:** Primer sequence used for real-time PCR analysis

Genes	Primer sequence (5′-3′)
Akt	F: ACGCTACTTCCTCCTCAA R: CTGACATTGTGCCACTGA
SOX9	F: AGCACAAGAAAGACCACCCC R: CGCCTTGAAGATGGCGTTAG
COL2A1	F: GCCAGGATGCCCGAAAATTAG R: GTCACCTCTGGGTCCTTGTTC
GAPDH	F: GGCAAGTTCAACGGCACAG R: CGCCAGTAGACTCCACGACA

### Western blot analysis

Cartilage oligomeric matrix protein (COMP) expression in cell pellets was analysed by Western blot at the end of week 3. Total protein was extracted and quantified using RIPA lysis buffer (Cwbio, China) and BCA protein assay kit (Applygen, China). Equal amounts of proteins were separated by 10% Sodium ﻿dodecyl sulfate-polyacrylamide gel electrophoresis (SDS-PAGE) and transferred onto a polyvinylidene fluoride (PVDF) membranes. After blocked with 5% non-fat milk in Tris-buffered saline tween-20 (TBST) for 2 h at room temperature, the membranes were incubated with primary antibody overnight at 4 °C. The primary antibodies used were rabbit anti-COMP (Abcam, USA) and anti-β-Actin (Affinity, USA). Next, the membranes were incubated with the secondary antibody of HRP-labeled goat anti-rabbit IgG (ZSGB-BIO, China) for 2 h at room temperature. Finally, the membranes were visualized with a super chemiluminescence western blot detection system (Applygen, China). Immunoreactive bands were quantified by densitometry using ImageJ software (NIH, USA).

### Immunohistochemistry (IHC)

The positive rate of the protein expressions of Akt in Wistar rat articular cartilage and subchondral bone were determined by IHC after Vaspin intraperitoneal injection for 14 days. The sections were dewaxed in xylene and dehydrated with gradient ethanol. After antigen retrieval in sodium citrate buffer (pH 6.0), the sections were placed in 3% H_2_O_2_ solution to deplete endogenous peroxidase. Next, the sections were blocked with 3% BSA for 30 min and incubated with anti-rat Akt antibody (Abcam, USA) overnight at 4 °C. Subsequently, the sections were washed three times in PBS and incubated with biotinylated IgG (ZSGB-BIO, China) at 37 °C for 30 min, followed by 3 times PBS rinse. Finally, dried sections were stained by freshly prepared diaminobenzidine (Solarbio, China) for 3 min to visualize the antibody-antigen complexes. Four typical fields (400 ×) were randomly selected from each section for observation and counted under a light microscope (Nikon, Japan). Positive reactions were defined as those showing brown or yellow signals in the cell cytoplasm. IRS system was used to quantify IHC staining. A staining index (values, 0–12) was determined by multiplying the score for staining intensity (0, no staining; 1, weak staining; 2, moderate staining; 3, strong staining) with the score for the proportion of positive cells (1, < 10%; 2, 10–50%; 3, 50–75%; 4, > 75%) [12].

### Statistical analysis

All data were analyzed by using GraphPad Prism 5.01 software (San Diego, CA, USA) and SPSS (Version 16.0, Chicago, IL, USA). Measurement data were presented as a mean ± SD. Comparison between two groups was conducted using independent-samples t test or separate variance estimation t-test (when the variance is heterogeneity). Comparisons among multiple groups were assessed by one-way analysis of variance (ANOVA). Values of *p* < 0.05 were considered statistically significant.

## Results

### The effect of Vaspin on the mRNA expression of Akt in chondrocyte differentiation

**Fig. 1:**
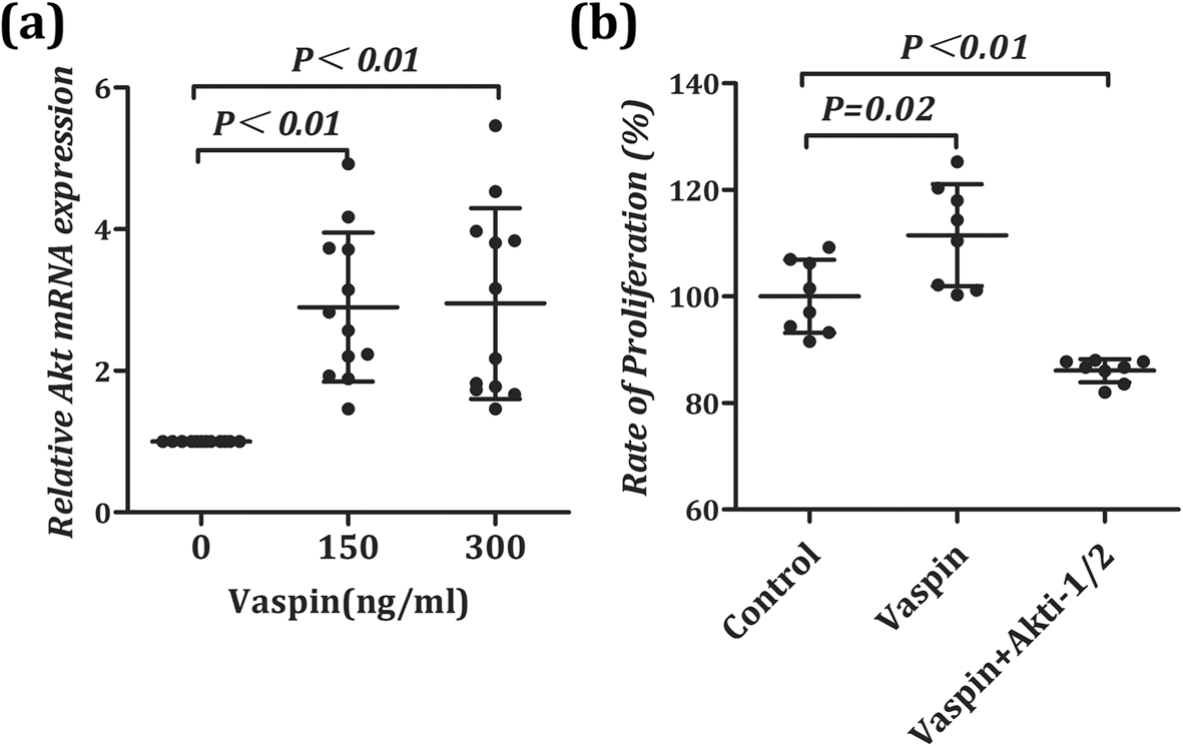
Vaspin contributed to chondrocyte survival by inducing Akt production. **a** Akt expression levels were measured by real-time PCR. Vaspin (150 ng/ml) treatment promoted Akt mRNA expression in chondrocyte. **b** The proliferation of chondrocyte after co-treatment with different gradient concentrations of Vaspin (0 ng/ml and 150 ng/ml) and Akt specific inhibitors API-2 (8 μmol/L) were analyzed by CCK-8 assays at 24 h. The results are represented the mean ± SD

To determine whether Vaspin plays a role by inducing Akt production in chondrocyte differentiation, we first detected the changes of Akt mRNA during BMSCs induced to chondrocytes after vaspin treatment with real-time PCR. We observed that the mRNA level of Akt in chondrocyte was significantly increased after Vaspin treatment (F = 15.19, *P* < 0.01) than that of the untreated chondrocytes (Fig. 1a).

### Vaspin contributed to chondrocyte survival by inducing Akt production

To determine whether there would be a potential impact of Vaspin on the survival of chondrocyte and whether Akt plays a role in the regulation of chondrocyte survival, we measured the change of chondrocyte proliferation ability after Vaspin treatment with a CCK-8 kit, and used Akt specific inhibitors API-2 as a control. The results showed that there were significant differences in the proliferative activity of chondrocytes among the three groups (F = 27.10, P < 0.01). We observed that there was a significant increase in the proliferation ability of chondrocyte after treatment with 150 ng/ml Vaspin (Fig. 1b, *P* = 0.02). However, the proliferation levels of chondrocytes were indeed inhibited after co-treatment with 150 ng/ml Vaspin and 8 μmol/L API-2 (Fig. 1b, *P* < 0.01). Together, these results indicate a potential role of Vaspin in chondrocyte survival. By blocking the production and activation of Akt, the above effects of Vaspin could be inhibited.

### The effect of Vaspin on the expression of different genes, involved in differentiation and ECM secretion of chondrocyte

**Fig. 2:**
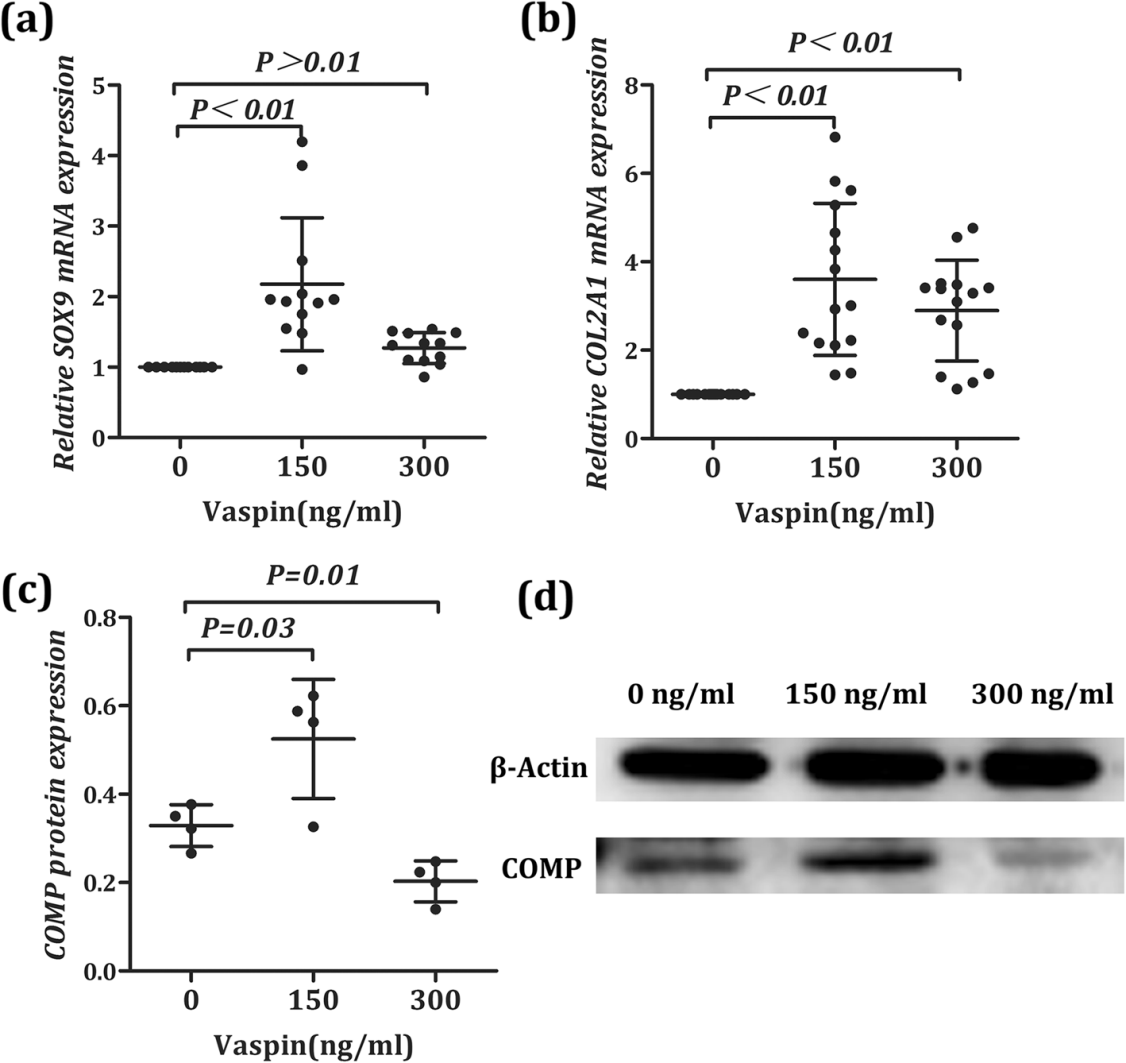
Effects of Vaspin on the expression of different genes, involved in differentiation and ECM secretion of chondrocyte. SOX9 mRNA (**a**), COL2A1 mRNA (**b**), and COMP protein (**c**, **d**) expression levels in chondrocyte were measured by real-time PCR or western blotting at different gradient concentrations of Vaspin (0 ng/ml, 150 ng/ml, and 300 ng/ml). The original blots in western blotting were showed in supplementary Fig. 2. Real-time PCR and Western blotting results are expressed as the mean ± SD

In order to determine whether Vaspin contributes to the differentiation of BMSCs into chondrocytes by affecting key genes expression in the process of chondrocyte differentiation and ECM secretion, we added Vaspin treatment in the process of inducing chondrocyte differentiation. After Vaspin treatment, the changes of key regulatory genes SOX9 in the process of chondrocyte differentiation and key molecules COL2A1 and COMP in the process of ECM secretion were detected. Firstly, we observed that after different concentrations of Vaspin treatment (150 and 300 ng/ml), the level of SOX9 and COL2A1 mRNA were significantly higher than that of the control group (F = 14.56, *P* < 0.01 and F = 21.05, P < 0.01), especially when the concentration of Vaspin treatment was 150 ng/ml (Fig. 2a, b, P < 0.01). Secondly, we found that in the process of cartilage induction and differentiation, after adding Vaspin with different concentrations, COMP protein expression level will show different change characteristics (F = 14.04, P < 0.01). Specifically, when Vaspin treatment concentration was 150 ng/ml, the expression level of COMP protein was higher than that of the control group (*P* = 0.03). However, when the treatment concentration of Vaspin was increased to 300 ng/ml, the expression level of COMP protein was lower than that of the control group (Fig. 2c, d, *P* = 0.01). The above research results suggested that Vaspin had a potential impact on the expression of different genes, involving in the differentiation and ECM secretion of chondrocyte.

### Vaspin promoted Akt expression in cartilage cavities and subchondral bone

**Fig. 3:**
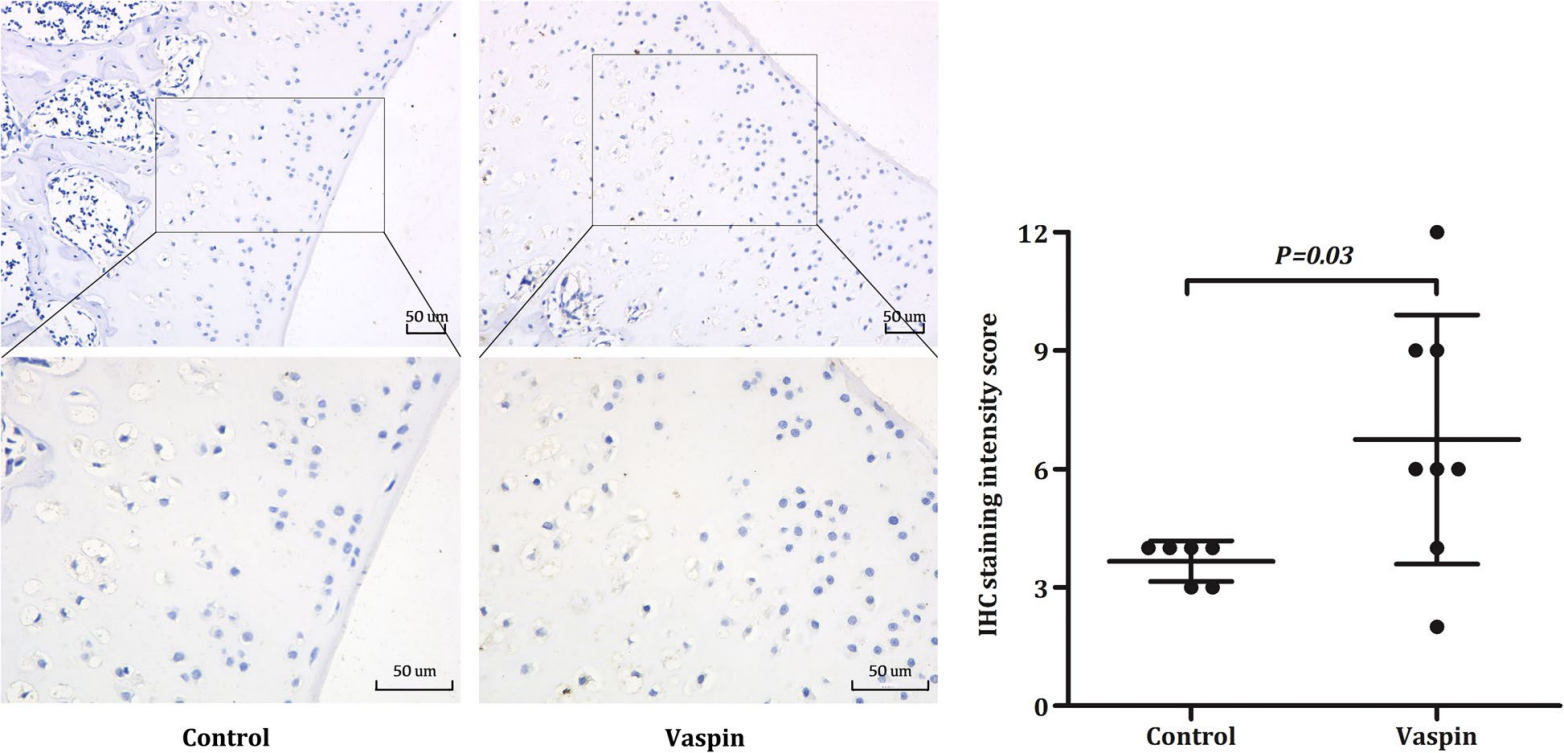
Vaspin promoted Akt expression in cartilage cavities and subchondral bone. IHC staining showing that positive expressions of Akt were significantly high after treatment with Vaspin (1 μg/kg/day for 14 days) in cartilage cavities and subchondral bone. Scale bar = 50 μm. The original IHC staining figures were showed in supplementary Fig. 1

To find the effect of Vaspin on the expression of Akt in cartilage and subchondral bone, we further evaluated the expression of Akt by semi-quantitative methods in Wistar rat articular cartilage and subchondral bone after Vaspin treatment by IHC staining. We observed that Akt was mainly expressed in the cartilage cavities and subchondral bone, and the protein levels of Akt were increased compared with control group after Vaspin treatment (Fig. 3, *P* = 0.03). Therefore, these observations might suggest that Vaspin was a promising candidate for affecting the internal balance of bone and cartilage via the Akt pathway in vivo.

## Discussion

It is well known that chondrocyte dysfunction and ECM degradation are the key factors in OA pathogenesis [13]. Therefore, attenuating chondrocyte dysfunction and ECM degradation have been proposed as a strategy for delaying the progression of OA. MSCs with self-renewal capacity and multi-differentiation potential have received much attention as an alternative approach in the management of chondrocyte dysfunction and ECM degradation in OA [14, 15]. Numerous studies have confirmed that chondrocyte differentiation and cartilage regeneration of MSCs are regulated by appropriate chondrogenic factors including adipokines [16–18]. Changes of systemic or local adipokines in OA will directly affect the metabolism of osteoblasts, osteoclasts and chondrocytes, which ultimately participate in the internal balance of bone and cartilage [8]. Vaspin is a typical adipokine that is widely expressed in a variety of tissues and cells, and participates in the internal balance of bone and cartilage by affecting the inflammation and metabolism of osteoblasts, osteoclasts and chondrocytes [19–21]. Our previous findings revealed that Vaspin is abnormally expressed in the OA bone marrow microenvironment and can affect the proliferation and survival of BMSCs in vitro [11]. However, whether Vaspin has a beneficial effect on the chondrogenic differentiation of BMSCs, and its underlying mechanism, there is no relevant report at present.

In the present study, we first demonstrated that Vaspin can promote the proliferation of chondrocyte, and the above effect can be inhibited by the Akt pathway specific inhibitor API-2. Although chondrocyte is a relatively inert cell and has little regenerative capacity, it still has the ability to proliferate in the early stage. Chondrocyte hypertrophy or senescence is thought to play a role in the initiation and progression of OA [22]. So our findings revealed that Vaspin might contribute to the survival of chondrocyte via Akt pathway, and could alleviate chondrocyte dysfunction and cartilage degeneration in OA progression.

MSCs or MSC like cells are believed to replace cells lost due to aging or tissue injury. ﻿Their intrinsic self-renewing ability and differentiation potential into chondrocyte, ﻿make them an attractive population of cells for ﻿cartilage repair in OA [15, 23]. A better understanding of the above processes is required to slow down or stop OA progression. Complex signaling networks have been suggested to mediate chondrocyte fate, inflammatory responses and ECM homeostasis. In particular, PI3K/Akt pathway is a key regulatory pathway for chondrocyte terminal differentiation [24], is also essential for maintaining cartilage homeostasis [25]. Firstly, the PI3K/Akt signaling pathway is a vital regulator of chondrocyte differentiation, survival and apoptosis, and PI3K/Akt activation can protect against OA via reducing chondrocyte apoptosis [26–29]. Secondly, the PI3K/Akt signaling pathway involves in the ECM homeostasis, activation of PI3K/Akt can promotes ECM anabolism and attenuate ECM catabolism [30–32]. In this study, we found that Vaspin could promote the transcription of Sox9, a key gene in the chondrogenic differentiation of BMSCs, and the transcription and expression of ECM-related genes COL2A1 and COMP. Sox9 is the most critical transcription factor in the process of chondrocyte differentiation and proliferation. From chondrocyte progenitor cells to end-stage chondrocytes, Sox9 is always actively expressed and is essential for the maintenance of chondrocyte lineages [33]. Collagen type II (encoded by COL2A1 gene) and COMP are important marker proteins of ECM in hyaline cartilage, produced and secreted in chondrocytes, are involved in the construction and maintenance of the ECM collagen fiber network, and play important role in the proliferation and activation of chondrocytes, as well as maintaining the normal permeability of the cartilage matrix [34, 35]. Therefore, our results suggest that Vaspin can promote chondrogenic differentiation of BMSCs and increase the ECM secretion in chondrocyte. In the above process, we also found that Vaspin could promote the expression of Akt in chondrocyte, a vital messenger in the PI3K/Akt pathway. PI3K/Akt pathway fulfills functions in many cellular processes essential for homeostasis, so our results suggest that Vaspin plays a role in promoting chondrogenic differentiation of BMSCs and stimulating ECM anabolism by activating the PI3K/Akt signaling pathway.

Subchondral bone, best known as the bony component lying under articular cartilage, supports cartilage and spreads mechanical forces across joint surfaces. Subchondral bone and cartilage form a functional complex called the bone–cartilage unit, which is involved in the pathophysiology of OA [36]. Over recent years, the role of subchondral bone in the pathogenesis of OA has gradually attracted increasing attention [37]. Microstructural alterations in subchondral bone might lead to cartilage destabilization, and further contribute to cartilage degeneration in OA [38]. PI3K/Akt pathway is a key metabolic pathway in subchondral bone. The activation of PI3K/Akt pathway in subchondral bone could promote osteoblastic differentiation in pre-osteoblasts and BMSCs and targeted inhibition of PI3K/Akt pathway could reduce the bone formation [39, 40]. Our immunohistochemical results showed that Akt was mainly expressed in the cartilage cavities and subchondral bone, and Vaspin could promote the expression of Akt in subchondral bone. These data provide evidence that exogenous and endogenous Vaspin could enhance local expression of Akt in subchondral bone, which might further promote the osteoblastic differentiation of BMSCs and had a certain value in attenuating the microstructural alterations in subchondral bone and delaying the progression of OA.

There were also some limitations in this study. Firstly, Akt, SOX9 and COL2A1 were only tested by qRT-PCR, and Western Blot was not performed for further verification. Secondly, p-Akt was indeed a hallmark of Akt activation. Although our previous study had confirmed that Vaspin could increase the phosphorylation of Akt, we did not further detect the level of p-Akt in this study. In the future, we will carry out more rigorously designed studies on the upstream and downstream genes of the PI3K/Akt pathway to further investigate the role of Vaspin in OA.

## Conclusion

Our data showed for the first time that Vaspin could promote the chondrogenic differentiation of BMSCs, and increase the proliferation and ECM secretion of chondrocytes through Akt activation. Furthermore, our study demonstrated that exogenous and endogenous Vaspin could promote the expression of Akt in subchondral bone, and involved in bone metabolism of subchondral bone. Vaspin might represent a promising agent to ameliorate the proliferation and chondrogenic differentiation of BMSCs in OA. Therefore, our study may lead to new insights into the potential effect of Vaspin in OA. Further investigations are warranted to explore the potential of Vaspin as a therapeutic target for OA as well as for other metabolic bone diseases.

## Supplementary Information

**Additional file 1 Supplementary Fig. 1.** The immunohistochemical staining results.

**Additional file 2: Supplementary Fig. 2.** The original blots in western blotting.

## Data Availability

All of the data in this study are obtained from experiments. The data used and analysed in this study are available from the corresponding author on reasonable request.

## Abbreviations

OA: Osteoarthritis
BMSCs: Bone mesenchymal stem cells
ECM: Extracellular matrix
CCK-8: Cell Counting Kit
qRT-PCR: Quantitative Real-Time Polymerase Chain Reaction
GAPDH: Glyceraldehyde-3-phosphate Dehydrogenase
COMP: Cartilage oligomeric matrix protein
SDS-PAGE: Sodium dodecyl sulfate-polyacrylamide gel electrophoresis
PVDF: Polyvinylidene fluoride
TBST: Tris-buffered saline tween
IHC: Immunohistochemistry
ANOVA: Analysis of variance
