# Supplementary material for: Vaspin promotes chondrogenic differentiation of BMSCs via Akt activation in osteoarthritis
Source: BMC Musculoskelet Disord. 2022 Apr 11;23:344. doi: 10.1186/s12891-022-05295-9 (PMC8996515; doi:10.1186/s12891-022-05295-9)
Supplement: Supplementary file 1 — Additional file 1 Supplementary Fig. 1. The immunohistochemical staining results. [file 12891_2022_5295_MOESM1_ESM.pdf]

Supplementary Figure 1. The immunohistochemical staining results.

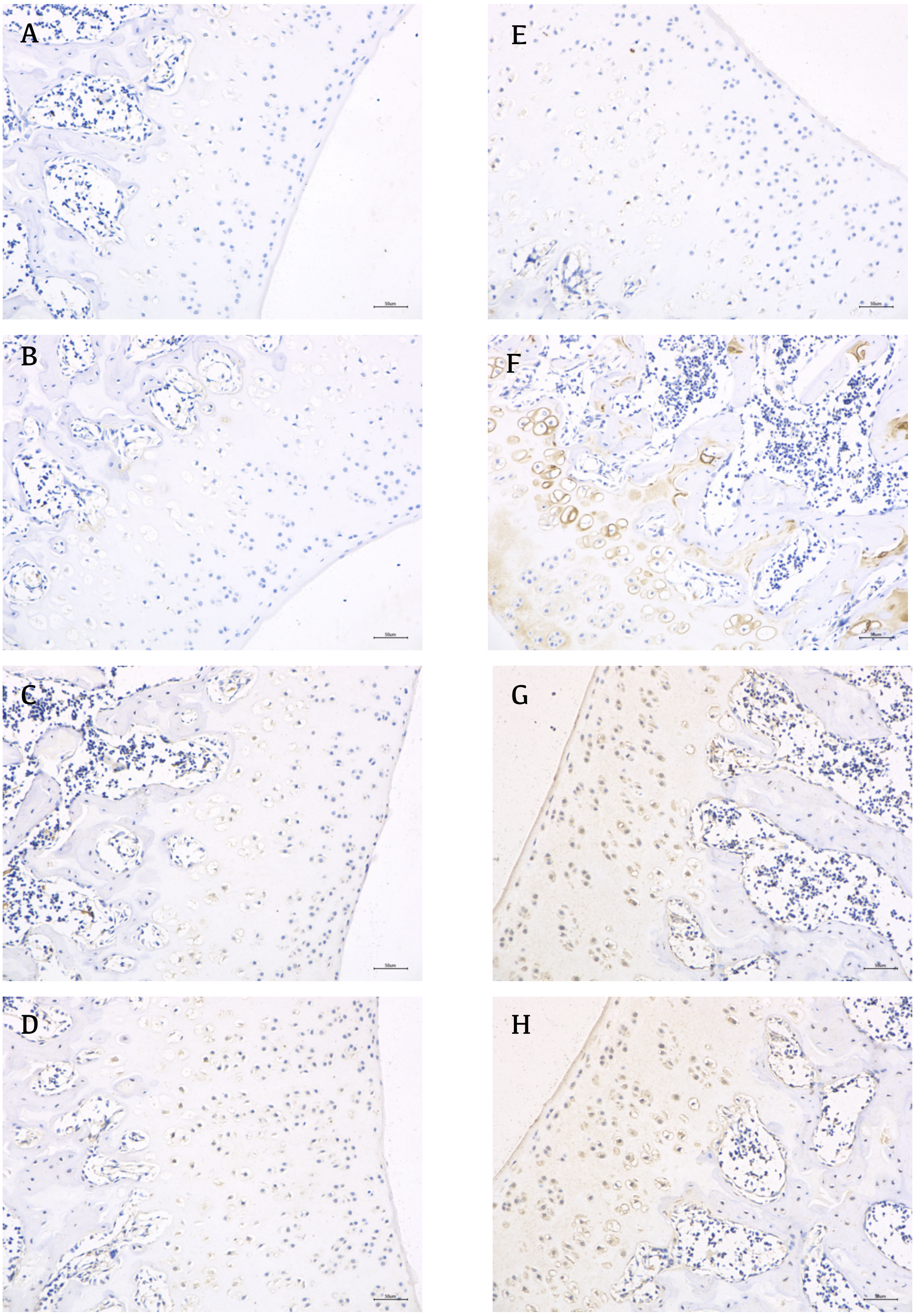

The protein expressions of Akt in Wistar rat articular cartilage and subchondral bone in Sham group (A-D) and Vaspin group (E-H).
