# Supplementary material for: Vaspin promotes chondrogenic differentiation of BMSCs via Akt activation in osteoarthritis
Source: BMC Musculoskelet Disord. 2022 Apr 11;23:344. doi: 10.1186/s12891-022-05295-9 (PMC8996515; doi:10.1186/s12891-022-05295-9)
Supplement: Supplementary file 2 — Additional file 2: Supplementary Fig. 2. The original blots in western blotting. [file 12891_2022_5295_MOESM2_ESM.pdf]

Supplementary Figure 2. The original blots in western blotting.

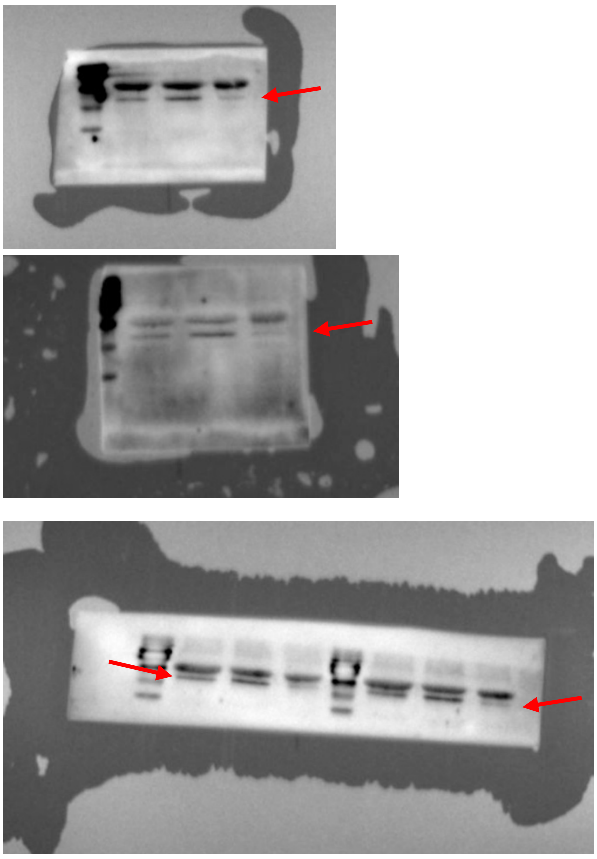

Above are the original blots of COMP.

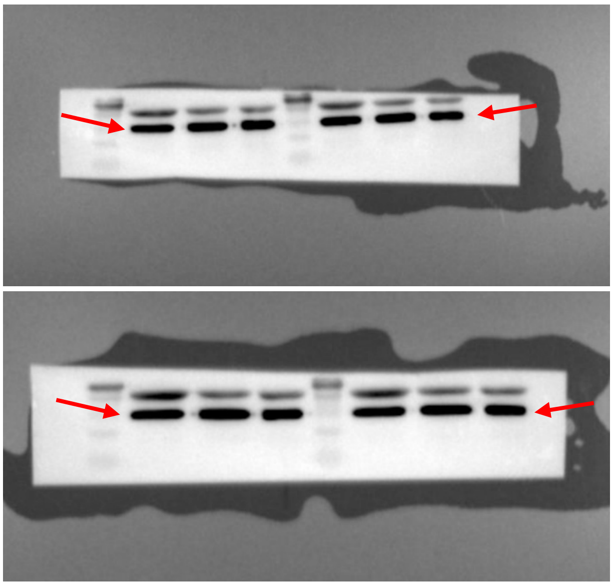

Above are the original blots of β-Actin.
